# Tumors of the Mouth

**Published:** 1882-04

**Authors:** M. B. Lowry


### ARTICLE IV.

### *Tumors of the Mouth.*

BY DR. M. B. LOWRY.

(Read before the Odontological Society of Western Pennsylvania )

Tumors of the mouth are of several kinds, simple hypertrophy of the gums, polypus, epulis, and vascular tumors, constitute the principal varieties.

#### HYPERTROPHY, OR MORBID GROWTH OF THE GUM.

The structural changes which take place in the gums, as a consequence of increased vascular action, are almost as various as are the constitutional tendencies of different individuals. Those characterizing this affection will be noticed growing up from about the teeth until they cover their crowns to a considerable extent. When thus affected the gums have a dark purple color, with thick, smooth and rounded margins, and discharge almost constantly a thin purulent matter, which has a very offensive odor. They bleed profusely from the slightest injury, and are also very sore. The lip pressing against them causes considerable annoyance and pain. They have also a peculiar itching sensation which is extremely annoying. Among the local and constitutional effects arising from this disease are offensive breath and vitiated saliva. The disease will also go so far as to destroy the alveoli, with loosening and ultimate loss of the teeth, enlargement of the tonsils and impairment of digestion, with all its disagreeable consequences and train of other phenomena.

*Causes*.—The exciting causes of this affection are local irritation, produced by tartar, necrosed alveoli, dead or diseased teeth, and sometimes a crowded condition of the teeth. Some constitutions are peculiarly susceptible of this affection, and will have it in its most aggravated form. I have never seen or read of a case where salivary calculus was not present acting as an irritant.

*Treatment.*—The first thing to be attended to is the removal of all foreign matter that does, or will in any way, act as an irritant. All morbid growths should be removed and incisions should be made so as to completely discharge the surplus blood. All foreign matter must be kept away, and the parts must be kept perfectly clean, and frequently lanced to discharge the morbid blood that may accumulate from time to time. In this way the progress of the disease may be arrested; but we must not expect a cure from local treatment alone. Particular attention must be paid to the regimen of the patient, as the particular nature of the case may indicate. During constitutional treatment, local measures must be continued, such as scarifying the gums, using mouth-washes of astringent solutions, keeping the mouth perfectly clean, etc., and you may expect a cure.

### POLYPUS.

This is a morbid excrescence developed from the mucous membrane of the mouth. *Polypi* are similar in appearance to the surrounding gum, but they differ widely in their texture, and in the nature of attachments. They vary much in size, mode of adhesion and intimate nature. Some are of a soft, fleshy, or gelatinous nature, springing from a narrow pedicle, widening out into somewhat of a pear or egg-shaped bulb. Some are wide at the base, extending between and around the teeth. Some are soft, fleshy, gelatinous, and others soft and fibrous. They are not generally vascular; but some will bleed freely on the slightest injury. They have received various appellations; as *mucous, soft, vascular.* The gelatinous and fibrous are not vascular, while others will bleed freely. There is seldom any pain attending this diseased tissue unless it receives some injury and ulcerates. It then may become very painful, and there will also be a discharge of thin, purulent matter which is very offensive. Under these circumstances, the patient may be unable to distinguish the pain from ordinary toothache; or from inflammation of a dental pulp. If allowed to take its own course, it will grow to a considerable size, encroaching on the tongue, and interfering with deglutition and respiration.

*Causes.*—The exciting causes of this affection are teeth that are decayed to or below the edge of the gum, with ragged sharp edges, a root, and necrosed or loose bone. We, at times, find it necessary to cut a V, or wedge shape between the teeth; and if proper care is not taken, there may arise a polypus between the teeth, the result of leaving a ragged edge, or sharp point, and perhaps crowding a small portion of the filling material along the side of the tooth, which may act as an irritant. In other words, *Polypus is produced by local irritation.*

*Treatment.*—The treatment consists in the early removal of the causes. This is done in various ways, according to the situation and nature of the tumor. If from a tooth decayed down to or below the gum, affording a receptacle for polypus, they are usually too deeply involved in disease to admit of successful treatment. Teeth and roots should be extracted and the tumor carefully cut away. All necrosed or loose bone must be removed and even a part of the healthy alveolus or gum. If from a defective operation, the operator must see to it that nothing about the tooth is left to act as an irritant. Cut the tumor from between the teeth. In my own practice I have used cotton saturated with sandarac varnish, dressing from time to time, and letting remain until all disposition of the tumor to return has been overcome. At times I use sulphate of copper in a powder form, for the cicatrization of the tumor.

### EPULIS.

This is a morbid growth, sometimes ending in cancer, *Odontia excrescence*, *Sarcoma epulis.* Tumors springing up from the margin of the gums, whatever may be their structural character, are in my opinion, Epulis. Both jaws are subject to this disease. The difference in structure depends somewhat upon the tissue in which they originate, as the gum, the membranes of the teeth, the periosteum of the alveoli, or the membranes lining the cavity. All the surfaces of the bone or lining of their cavities are subject to Epulis, they becoming the seat of tumors of every size and consistency, benign and malignant. We also find the tongue and lips involved in this disease.

In many cases it may be extremely difficult, if not impossible, to find any satisfactory cause for the occurrence of epulis; but in others, an examination of the tumor reveals a source of the disease, or rather irritation to which the presence of the disease may be assigned. The disease, at its outset, is usually confined to the edge of the gum, and, as a general thing, that portion which lies between the teeth. The attachments may be by a small and flattened pedicle, or by a broad base. The tumor is generally of slow growth. It may grow till it covers the whole of the alveolar ridge, forcing the teeth from their sockets, encroaching on both hard and soft palate, interfering with mastication and deglutition. The tumors springing from the fibrous tissue are very generally themselves fibrous in character. In respect to vascularity they generally correspond with the adjacent gum, and the density of the tumor.

When the tumors have attained considerable size, they become malignant, which very much alters the character of the disease. When the surface is injured, the injured part becomes the seat of ulcers, which emit a copious and fetid discharge and the patient has acute suffering and great annoyance; when malignant they are vascular, and hemorrhages are liable to occur. Epulis will show hues varying in color from a deep red to a buff, and a peculiar light greenish tint of yellow.

Epulis when benign is generally of a red color, and a soft and spongy consistence. It is a smooth, shining tumor, elastic, compressible, but little sensitive, and will bleed freely when cut. The disease may originate in the sockets of teeth that appear perfectly sound, and may be accompanied with swelling and a discharge of pus. We have another form which is extremely sensitive accompanied with acute lancinating pain. We also have the cancerous, or malignant tumor.

*Treatment.*—The treatment of Epulis will depend upon circumstances. If the tumor is benign, depending on irritation alone, caused by decayed teeth, roots, etc., it is only necessary to remove the cause of the trouble, and then cut away the tumor, and destroy what may remain of it by caustic or by compression. If the tumor be of the elastic kind, it will require a thorough removal. If attached by a narrow neck, it can be removed by a ligature or the knife. If the latter is used, profuse bleeding may follow, requiring the use of persulphate of iron or the actual cautery. If the tumor is of scirrhus form, a very thorough excision will be required. All bony structures in any way connected with it, must be boldly cut away, and such other treatment adopted as the circumstances may demand. Whatever the nature of these tumors, they should be carefully watched, and on the slightest indication of a return of the disease, prompt treatment must be adopted, with the knife, caustic, or actual cautery, as the nature of the case may require.

### FISTULOUS TUMORS.

I will here mention a case of a fistulous tumor. A lady came to me last June for treatment. She had a tumor on the left side of the inferior maxilla, extending from the second bicuspid as far back as the wisdom tooth. It was in shape something like a cauliflower, and, of a rather light pinkish red color. Its texture was of a rather solid, fleshy consistence with a slight discharge of pus. The tumor had grown up to interfere with mastication, but was not painful.

*Causes.*—The tumor originated from a blow received on the side of the jaw, breaking a small portion of the alveolus.

*Treatment.*—I cut away the tumor, removed all the broken alveolus, syringed the parts clean with a solution of one part carbolic acid to nine of water. The gums healed almost by first intention. After the removal of the tumor I cut it open, found a sack containing thick pus, the tumor also contained a small portion of loose alveolus.

OSSEOUS TUMOR.

I will also speak of a lady who came to me a few years ago for treatment of exostoses of both superior and inferior maxillaries. The growth covered the whole of the alveolar ridge of both jaws, causing the lips to protrude to a considerable extent. There was no pain attending the disease. The growth was near one-half inch in thickness, with the gum growing tight over the tumor.

*Cause.*—I could never find any cause other than tartar, of a very dark and hard consistency, attached firmly to the necks of the teeth.

*Treatment.*—After extracting all of the teeth, I dissected the gum below the osseous growth, and, with excising forceps, chisel and file, cut the whole of the growth away, trimmed the gum to make a complete union, and the parts healed nicely. I never saw any disposition of the disease to return, while watching it for three years. At the expiration of one year, I inserted a full set of teeth, which proved very satisfactory.—*Ohio State Journal.*

